# Social Skills and Cognitive Training to Support Work-Related Skills and Job Placement in a Group of Autistic Adults: Effectiveness of a Neuropsychological and Social Skills Intervention: A Case Series Study on a Pilot Program

**DOI:** 10.1007/s10597-023-01152-8

**Published:** 2023-06-15

**Authors:** S. Brighenti, L. Mustacchia, G. Cicinelli, S. Chieregato, C. Comella, L. Torrero, F. Granata, R. Keller

**Affiliations:** 1 Adult Autism Center, Mental Health Department ROT NO, Local Health Unit ASL Città di Torino, Turin, 10138 Italy; 2 Emilio ETS, Via Vittorio Amedeo II, 17, Turin, 10121 Italy; 3 Consorzio Abele Lavoro, Via Paolo Veronese, 202, Turin, 10148 Italy; 4 Department of Psychology, University of Turin, Via Giuseppe Verdi, 8, Torino, TO 10124 Italy

**Keywords:** Autism, Work, Job placement, Neuropsychological training, Social skills training

## Abstract

Autistic people may have difficulties in finding and keeping a job. Studies highlight that only 34% of autistic people are employed compared to 54% of people with disability. 58% of people with ASD have never had a job. Social cognition and cognitive strains may also have a significant impact on working life. The primary goal of our project is supporting autistic people through a training program focused on neuropsychological and social skills training to improve participant’ job skills. Through an Individual Placement and Support model the project involved various Partners to guide, identify skills and interests, provide cognitive and psychological support for autistic people. Results highlighted neuropsychological training efficacy, especially in inhibitory control and good rate of employment status at the end of the project. Findings are encouraging and underline the importance of a multidisciplinary approach to support autistic people in their work life considering their expectations, needs and inclinations.

## Introduction

Autism Spectrum Disorders (ASD) are neurodevelopmental disorders characterized by persistent deficits in communication and social interaction, restricted and repetitive patterns of behavior, interests, or activities and sensory hypo or hypersensitivity (APA, 2013). Autism can be defined as a neurodiverse way of facing difficulties of adaptation in working contexts. Adults with ASD find themselves facing various trials throughout their life and, one of the most difficult is finding a job (Hillier, et al., 2007).

Recently, job placement for autistic people has been a matter of great interest from research and from public and private institutions due to the economic impact of unemployed people on public spending. In Western countries, ASD adults’ labor force participation rate is 34% compared to 54% of people with disabilities and 83% of people without disabilities (Howlin, et al., 2004; Howlin & Moss, 2012). An American study from Drexel University reports that young adults with ASD have the lowest employment rate compared to other disabilities. Indeed 58% of people with ASD have never had a job (Roux, et al., 2017).

Finding and maintaining a job is demanding for ASD people, even with or without cognitive lack (Frank, et al., 2018). Additionally, follow-up studies of employed people have found that the majority of jobs held by people with ASD are unskilled and poorly paid. They are typically part-time employed and work an average of less than thirty hours a week (Baldwin, et al., 2014). In their research, Muller and colleagues (2003) interviewed ASD people and as a result they evidenced long periods of unemployment and/or under-employment. Consequently, this job status leads to smaller opportunities for career advancement.

In Italy, in order to look for a job, the Law n°68/1999 established the Targeted Placement, a legal arrangement that allows people to obtain support from the State. This law applies indistinctly to people with disabilities including ASD people. Moreover, it allows companies to employ people who benefit from Law n°68/1999. Nowadays, the employability rate of people with disabilities available in Italian context also includes autistic people. Only 31.3% of people with disabilities aged between 15 and 64 are employed compared to 57.8% of people without disabilities, in the same age range. Furthermore, females with disabilities are more disadvantaged: only 26.7% of women with disabilities are employed compared to males’ rate of 36.3% (Istat, 2017).

Research suggests that cognitive and social interaction difficulties as well as core symptoms are barriers to career success for ASD people (Brighenti, et al., 2018; Hillier, et al., 2007; Baker-Ericzén, et al., 2018).

Cognitive functioning, especially executive functions and social skills are described as “soft skills” in work contexts, and it has been highlighted that deficits in these areas create difficulties in finding and keeping a job (Hillier, et al., 2007; Mawhood and Howlin, 1999). Establishing and maintaining a conversation, communicating one’s needs, and interpreting facial expressions are several social skills difficulties that autistic people can face in the workplace (Chen, et al., 2015). Few studies consider both neurocognitive and social skills training to enhance employability of ASD people. A pilot study from Baker-Ericzén and colleagues (2018), despite a small number of people, found employment rates doubled after the intervention, with an increase from 22 to 56%.

The traditional model for job placement is the “*Train and Place*” (Jäckel, et al., 2017; Corrigan & McCracken, 2005). This model provides preliminary training before job placement; the effectiveness of this model is estimated at 30% in terms of employability (Marshall, et al., 2014). Recent studies demonstrated greater effectiveness of a new model of job placement, called “*Place and Train*” (Wehman & Moon, 1988, Corrigan PW, McCracken SG. 2005), consisting of simultaneous job placement and training, from which the Individual Placement and Support model origins (IPS; Becker and Drake, 2003; Swanson & Becker, 2011; Fioritti and Berardi, 2017). The IPS model, which inspired our project, aids people in finding a job in a relatively short time. Additionally, based on the characteristics of the person, individualized training and job-related support is provided (Wehman & Moon, 1988) to allow people to adequately keep the work. Rather than a preliminary training (as in “*Train and Place*” paradigm), a joint support to the company and the worker through a series of specific adaptations for the person and support actions is given both inside and outside the company (Marchisio & Curti, 2019).

Latimer et al. (2006) showed that with the IPS model results are more than double in terms of job placements on the competitive market compared to the “*Train and Plac*e” method.

## Methods

### Participants

The project was implemented in Italy, in the City of Turin, Piedmont Region. A specifically job-placement team was involved in the projects. Participants were recruited from the Regional Adult Autism Center and supported by 2 psychologists. For all participants a diagnostic assessment according to Multistep Multinetwork Model (Keller, et al., 2020) was provided. Other involved partners were Abele Lavoro Social Consortium and the Emilio ETS. 10 people with ASD, 2 females and 8 males aged between 21 and 37 years (see Table 1) were involved. 9 people were diagnosed with ASD level 1 (according to DSM-5) with Intelligent Quotient (IQ) > 70 and 1 with ASD Level 2 (according to DSM-5) with IQ < 70 and speech impairment.

**Table 1:** Descriptive characteristics of the sample (n = 10)

	Age	Education	FSIQ
M (SD)	29.7 (5.81)	13.5 (2.84)	78.8 (13.9)
Min	21	8	58
Max	37	18	102

**Fig. 1:**
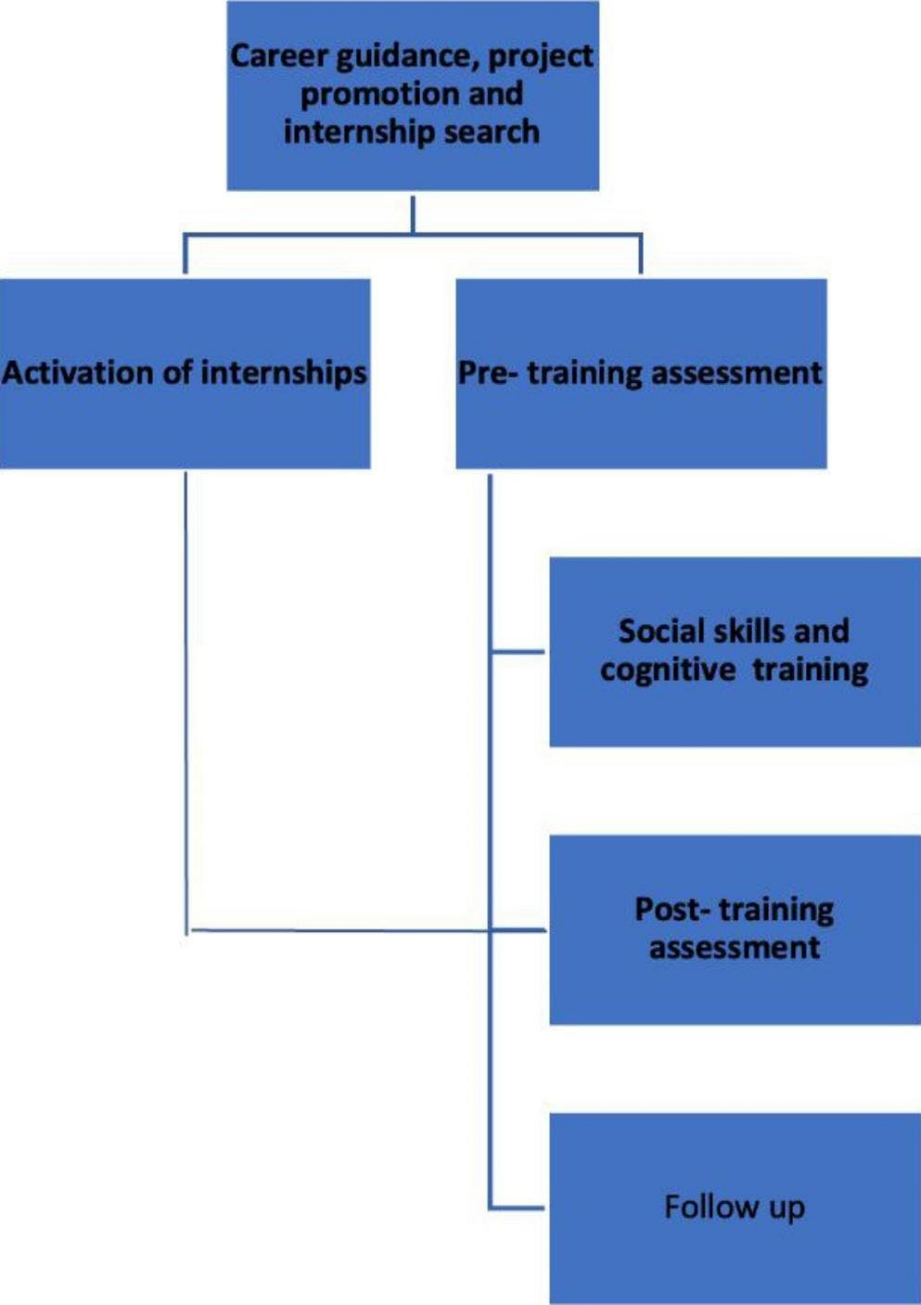
Project phases

### Phases of the Project

#### Career Guidance, Project Promotion and Internship Search

The project involved several phases (see Fig. 1). At the beginning, participants were involved in group meetings organized by one of the Partners (Consorzio Abele Lavoro). These groups were conducted by two counseling psychologists and aimed to identify one’s own skills and professional interests. Participants were trained for finding job ads aligned both with Law n°68/99 and people’ curricula.

Individual interviews aimed at deepening participants’ professional profile and reviewing their curriculum vitae were also led.

In order to identify the host companies, promotional activities for the project were carried out.

#### Activation of Internships

Based on the available budget, cross-referencing between companies and participants’ work-related skills was carried out to activate 5 paid internships. The evaluation of the suitability of companies was made through several actions: visits to the company and telephone contacts with tutors. Meetings with the Adult Autism Center team and with family members were part of the main activities for supporting participants during the internships. In collaboration with the other project partner, Emilio ETS Association, educational and autism ‘awareness sessions were held with employers and tutors in the traineeship companies.

#### Pre-training Assessment

Subsequently, to structure group training, neuropsychological, psychological, and behavioral assessments were carried out (see below for details).

#### Cognitive and Social Skills Training

At the end of the assessment phase, the group activities were set up. The groups’ goals were cognitive enhancement and job-related social skills improvement.

To support people in maintaining and better performing in their internships, group training lasted throughout the entire project. A large room of the Adult Autism Center with a table and a whiteboard for taking notes was used for the group meetings.

Additionally, individualized, and personalized interventions were activated on participants’ request or if needed for the entire duration of the project.

18 cognitive training sessions, lasting 90 min each, took place on a weekly basis. Due to the limitations of Covid-19, two groups of 3 participants and one of 2 were done. The cognitive training was led by a neuropsychologist.

The cognitive training sessions goals were formulated considering the results of the initial neuropsychological evaluation. During the sessions a combined approach between restorative and compensatory methods (Làdavas, 2012) was used. Exercises were individual or group based. Moreover, homework was given to generalize the abilities. Enhancement of the participants’ metacognitive skills was one of the main objectives. Thus, we expected people to find strategies to compensate for their cognitive difficulties.

During the meetings, ASD cognitive functioning was deepened, helping participants to choose their own facilitating tools (i.e., diaries, calendars, alarm clocks, photos, videos, blackboards, notes) and find strategies for adapting to one’s living or working environments.

Aims pursued through the cognitive training were:

- improvement of attentional functions (auditory and visual attention; selective and divided attention, shifting abilities).
- improvement of executive functions (working memory, verbal fluency, planning and problem-solving, flexibility and inhibitory control).
- enhancement of memory functions (short-term memory, coding, storage, and retrieval strategies).

Social skills training groups were conducted twice a week for a total of 10 meetings lasting 75 min each. Two groups with 4 participants were set up. Training was led by a cognitive-behavioral psychotherapist.

In the social skills training groups, the Behavior Skills Training model (BST; Sarokoff and Sturmey, 2004), specifically designed to teach new skills, was used. The BST model consists of 4 different steps: Instruction, Modeling, Practice and Feedback. The skills trained were partly chosen by the conductor based on the assessment and partly requested by participants since their difficulties experienced in the internships or in social life. The covered topics were face to face interview, introducing yourself, starting, maintaining, and ending a conversation, sharing breaks with colleagues, joining a group, leaving a group, expressing assertive critics, expressing unpleasant feelings.

8 participants were included in both groups (cognitive and social skills training). Due to personal issues one participant attended 8 online psychological sessions with the psychotherapist. 29 individualized cognitive-habilitative training of two hours a week was conducted for one participant with cognitive and speech impairment.

#### Post-training Assessment and Follow-up

At the end of the training sessions, a retest phase was run. For collecting participants’ feedback, a qualitative questionnaire was also administered. After 5 weeks, a phone-based follow-up was carried out to check the prosecution of internships, the activation of new work experiences or job searching.

### Assessment Tools

For cognitive assessment, the Repeatable Battery for the Assessment of Neurological Status (Randolph, 1998; Ponteri, et al., 2007) was used. The RBANS is a short screening battery composed of two parallel forms for the main cognitive functions which allow the evaluation of the effectiveness of cognitive training. The administration of RBANS is helpful to quantify the neuropsychological functioning in various areas. The performances are measured in terms of Indices for five cognitive domains: Immediate Memory (RABANS IM), Delayed Memory (RBANS DM), Visual Spatial/Constructional (RBANS VC), Language (RBANS LN), Attention (RBANS AT) and a Full-Scale Indices (RBANS FS). The Index scores have a range that varies from 40 to 160 and a mean equal to 100 with a standard deviation equal to 15. A performance below 2 SDs is considered below the norm, i.e., a score equal to or lower than 70 in terms of percentile ranks, a performance below the 5th rank is considered a deficit. A performance between the 5th and 25th percentile corresponds to a performance at the lower limit; between the 25th and 75th percentile a performance in the range of medium variability (with average at the 50th percentile), a performance above the 75th percentile is placed at a medium-higher level.

To assess executive functions, the Frontal Assessment Battery (FAB - Appollonio, et al., 2005; Dubois, et al., 2000) was administered. The FAB is a short screening battery and is useful for quantifying categorization skills, verbal fluency, executive planning, inhibitory control, and sensitivity to interference. Age and education are used for adjusting and transforming raw scores (FAB RS) in adjusted scores (FAB CS) (Apollonio, et al., 2005).

To quantify cognitive difficulties in everyday contexts, the Italian version of Cognitive Failures Questionnaire (CFQ; Salmaso, et al., 1988; Broadbent, et al., 1982) was used. The total score indicates the percentage (maximum value = 100) of cognitive difficulties in everyday life.

The Adaptive Behavioral Assessment Scale-II (ABAS-II; Harrison and Oakland, 2003; Ferri, et al., 2014) was administered in the self-report form. The ABAS-II is a questionnaire that provides a global and daily functioning assessment of people in different life contexts. It is used to assess strengths and weaknesses and to monitor interventions over time. A scaled score of 10 ± 2 represents the mean. The ABAS-II includes subscales for communication, community use, functional academics, home living, health and safety, leisure, self-care, self-direction, social, and work. Four composite scores are derived from the sum of the scaled scores: general adaptive (GAC), conceptual (DAC), social (DAS), and practical (DAP) composite scores.

The Social Responsiveness Scale - Second Edition (SRS-2; Constantino and Gruber, 2012; D’ardia, et al., 2021) is a 65-item scale that assesses the severity of symptoms and behaviors frequently associated with autism. In addition to the total score (SRS Tot), 2 other DSM-5-related scales can be calculated: Social interaction and communication (SCI) and Restricted and repetitive behaviors and interests (RRB). Furthermore, there are 4 treatment subscales: Social Awareness (AWR) as the ability to pick up on social cues, Social cognition (COG) as the ability to interpret social cues once they have been grasped, Social Communication (COM) that includes expressive social communication; social Motivation (MOT) as how the subject is generally motivated to engage in social-interpersonal behavior and Restricted Interests and Repetitive Behavior (RBB) that includes stereotyped behaviors or highly restricted interests.

Scores equal to or lower than 59 T are considered within the normal limits, scores between 60 T and 65 T indicate a mild deficit in reciprocal social behavior, scores between 66 T to 75 T indicate a moderate deficit in reciprocal social behavior and scores over 76 T or greater indicate a severe deficit in reciprocal social behavior.

To assess depressive symptomatology, the Beck Depression Inventory (BDI-II; Beck, et al., 1996; Ghisi, et al., 2006), a self-assessment tool consisting of 21 multiple-choice items, was used. The questionnaire measures the severity of depression in adults and adolescents aged 13 and over. Total score between 0 and 13 indicates a minimal level of depressive symptoms, between 14 and 19 is mild level of symptoms, between 20 and 28 moderate, between 29 and 63 severe.

The State-Trait Anxiety Inventory (STAI-Y; Spielberger, 1989; Pedrabissi and Santinello, 1989) questionnaire was used to assess state anxiety (form Y1) and trait anxiety (form Y2). Higher scores are positively correlated with higher levels of anxiety.

In addition, an assessment tool containing a list of the person’s work skills (adapted from Panisi and Keller, 2019) and his/her sensory functioning profile was filled in for each participant in order to offer to the hosting companies’ useful indications about the needs and characteristics of people.

Eventually, to collect the participants’ general feedback about the projects a qualitative questionnaire was used. Through this, it was possible to receive information about the general experience, internships, tutoring and support and the cognitive enhancement and social skills training activities directly from participants.

## Results

Jamovi Suite (The jamovi project, 2022) was used for the statistical analysis. We ran a paired sample t test with Wilcoxon’s ranks. Statistically significant results were found in FAB inhibitory control W (0.00) p < 0.049 and RBANS Visual Spatial/Constructional Index: W (8.00), p < 0.049. Table 2 (in appendix) shows the results obtained by each participant.

**Table 2:** Raw, scored and T scores for each participant for each test

Scales		1* T0 |T1	2* T0 |T1	3* T0 |T1	4* T0 |T1	5* T0 |T1	6* T0 |T1	7* T0 |T1	8* T0 |T1	9* T0 |T1	10* T0 |T1
SRS-2	AWR	58|75	58|58	71|58	52|58	64|58	48|51	68|48	65|61	65|51	41|44
	COG	76|83	68|58	74|70	61|70	65|53	50|64	61|54	67|70	65|72	61|59
	COM	77|84	57|65	70|63	67|70	63|59	84|84	54|53	90|90	75|76	54|54
	MOT	68|62	56|60	66|56	66|64	76|62	83|68	51|51	85|89	64|72	55|53
	RRB	54|70	64|60	66|68	70|71	66|56	45|50	43|43	89|73	73|73	50|45
	SCI	76|83	61|64	74|65	66|71	69|60	74|74	57|52	90|90	72|74	55|54
	TOT	72|80	62|63	73|66	67|72	71|59	68|69	53|50	90|90	70|75	54|52
ABAS II	GAC	16° − 2°	42° − 18°	0.3° − 1°	8° − 19°	23° − 34°	1° − 1°	9° − 23°	4° − 2°	27° − 30°	13° − 13°
	DAC	14° − 14°	53° − 37	0.5° − 1	12° − 21°	53° − 45°	2° − 2°	12° − 12°	12° − 14°	25° − 25°	18° − 14°
	DAS	10° − 3°	19° − 7°	1° − 3°	14° − 10°	7° − 14°	0.3° − 0.3°	3° − 21°	1° − 0.5°	14° − 34°	27° − 27°
	DAP	21° − 8°	42° − 16°	2° − 4°	10° − 25°	12° − 39°	5° − 3°	18° − 47°	13° − 5°	45° − 27°	9° − 9°
RBANS	RBANS IM	13° − 15°	57° − 23°	0,2° − 0,2°	8° − 0,3°	33° − 83°	0,2° − 12°	0.3°.-2°	79° − 57°	0.2° − 9°	0,2° − 6°
	RBANS VC	0,1° − 0,2°	63° − 3°5	0,3° − 0,8°	9,9° − 63°	63° − 83°	9,9° − 17°	0.2° − 0.3°	4° − 4°	0.2° − 0.2°	0,3° − 63°
	RBANS LN	13° − 15°	23° − 19°	0,3° − 0,3°	31° − 23°	44° − 48°	19° − 27°	0.2° − 4.2°	9° − 9°	23° − 2°	0,3° − 4°
	RBANS AT	36° − 4°	0,2° − 0,8°	0.1° − 0,2°	0.1° − 0.1°	8° − 13,8°	0.2° − 0.2°	0.1° − 0.1°	8° − 21,5°	0.1° − 0.1°	9° − 0.3°
	RBANS DM	23° − 0,3°	36° − 59°	0,2° − 0,1°	6,3° − 2,3°	31° − 52°	15° − 0.3°	0.3° − 0.2°	67° − 40°	0.2° − 36°	8° − 67°
	RBANS FS	0,3° − 0,3°	29° − 9,9°	0,2° − 0,2°	0,3° − 0,3°	21° − 61°	0.3° − 0.3°	0.2° − 0.2°	17.9° − 8°	0.1° − 0.3°	0.3° − 8°
CFQ		50 − 46	39 − 37	-	47 − 32	34 − 32	52 − 40	15 − 13	51–51	51–65	41 − 40
FAB RS		14–16	15–15	10–10	17 − 16	18–18	14–17	11–11	15–17	15–15	16–17
FAB CS		12,19 − 14,19	14,87 − 14,87	8,5–8,5	15,7–14,7	18–18	13,3–16,3	9,7–9,7	13,9–15,9	13,9–13,9	14,4–15,4

*1 = Internship + Group training; *2 = Online psychological sessions with the psychotherapist; *3 = Individualized cognitive-habilitative training; *4 = Internship + Group training; *5 = Internship + Group training; *6 = Group training; *7 = Internship + Group training; *8 = Group training; *9 = Group training; *10 = Internship + Group trainin

As a result of the internship phase one participant worked in a retail company as a shelf operator; another one worked in a small organic shop dealing with both sale and the organization of the shop (shelves, cleaning); another participant worked the internship at a farmhouse taking care of keeping the spaces in order; one participant completed an internship with office duties in an association and another participant attend his internship at a sportswear company but was unable to complete the hours set for personal reasons. Thus, the adherence rate to the planned paid traineeship courses was 80%.

At the end of the course, a feedback questionnaire on a 5-point Likert scale was administered. It made possible to collect the direct opinions of the participants, thus capturing the strengths and weaknesses that could be improved about the project. Below we indicated the average scores of the questionnaire.

Considering all the internships (5 internships included and two found outside the project) 70% of the participants carried out an internship. All the participants believe that the activities during internships were in line with their personal aptitudes and provide a very positive evaluation of this aspect (4.6/5). The judgment of the traineeship locations was very good (4/5) in terms of the journey to reach the place (4.6/5), moderate in terms of work time (3.5/5), type of activities carried out (3. 5/5) and regarding the overall duration of internships (3/5).

Good feedback in terms of matching of the internship with their needs, interests, and desires (3.7/5) was given.

The participants also evaluated positively the activities carried out during the internship (3.8/5) and the relationship with work colleagues (4.2/5), as well as the tutoring received (4.2/5).

The project was judged useful from all participants for their own personal training. Specifically, the sub-dimensions investigated were:

- general interest in the activities included in the project (classroom training, group activities, internship) with respect to personal empowerment (3.7/5).
- coherence between the project and one’s own needs: (3,44/5)
- satisfaction with the project: (3,77/5)
- completeness of the project: (3,5/5)
- formativeness: (4/5)
- utility of the project: (4/5)

The project experience was not judged as stressful (2/5). The overall commitment required was judged as adequate (3.2/5) and the course as adequately motivating (3.6/5).

As regards to the cognitive enhancement activities, these were perceived by the participants as interesting (3.9/5), in line with their needs (3.8/5) satisfactory (3.8/5), complete (3.3/5), educational (3.9/5), motivating (3.7/5) and useful (4/5). In terms of perceived stress, they were evaluated as not very stressful (1.6/5) and adequate in terms of effort required (3/5).

The Social Skills Groups were judged interesting (3.8/5), in line with the needs of the participants (3.6/5) and complete (3.2/5). They were judged very educational (3.7/5), motivating (3.6/5) and useful (3.8/5). They were judged low stress (1.9/5) and almost adequate in terms of commitment required (2.7/5).

The most positive reported aspects of the project were the internships, cognitive and social skills training (70%), the overall organization and tutoring during the internship (50%), the individual support received and the group activities (40%). 20% of the participants indicated the pre and post training assessment as negative aspects of the project.

After 5 weeks from the end of the project participants were called to get feedback on their employment status.

Of the 5 people chosen to carry out the internship within the project:

- 1 was hired by the same company of the internship with a fixed-term contract of 6 months (indicated as participant n° 5 in Tables 2 and 3).
- 1 was hired by a company that deals with data analysis (same job performed during the internship) and will soon sign the fixed-term employment contract (indicated as participant n° 8 in Tables 2 and 3).
- 1 is still carrying out the internship thanks to a 6-month extension.
- 1 is carrying out a training course in an area similar to the internship (sales employee).
- 1 is not currently looking for work.

**Table 3:** Job path and outcomes by phases for participant 5 and 8

	Participant n°5	Participant n°8
**Participants selection** October 2021	Unemployed, not looking for a job. No rehabilitation activities during weekdays.	Unemployed, looking for a job. Daily activity: gym.
**Phase 1** November-December 2021	Assessment of the interests, abilities, motivation, and previous experiences analysis.	
	Interests: care of green areas, gardening, stockkeeping. Previous experiences: several obstacles with colleagues and tutors due to socio-emotional difficulties.	Interests: administrative duties. Previous experiences: short successful short-term job. Weaknesses: executive functioning (e.g., planification and organization).
**Phase 2** January 2022	Activation of the healthcare professionals’ network including neuropsychological and social skills trainings.	Neuropsychological and social skills trainings.
	Job opportunity: sales company that allow the participants to go outside of retail hours so without social contact. Type of contract: 6 months fixed term.	Job opportunity: no-profit company Type of contract: 6 months fixed term.
**Phase 3** December 2021 – January 2022	Pre-training assessment See Table 2 for scores	
**Phase 4** January – June 2022	High participations to training sessions, especially the social skills training group because the participants used the session to analyze and better understand emotional and social challenges with colleagues and tutors during the internship.	High participant and motivation, especially for the neuropsychological sessions. The participant wanted to improve his ability to organize and plan things to get greater autonomy at work.
**Phase 5** June 2022	Participant’s contract was extended for other 6 months	The internship ended and the participant started actively to look for a job
**Follow—up** September 2022	The participant is still working in the same company, and he is satisfied with the job and the relationship with colleagues.	The participant signed a 6 months fixed-term contract in a company whose field is like the one he worked in during the internship.

In Table 3 is summarized the job path of the first two participants out of five people chosen for the paid internship because in our opinion they had the strongest motivation in the project.

Of the remaining participants that did not complete the internship within the project:

- 1 is actively looking for a job with the help of an educational figure who supports him in writing and applying for several positions.
- 1 is carrying out an internship outside the projects and is actively looking for volunteering activities simultaneously.
- 1 works part-time in the family business.
- 1 is actively looking for job with the help of the employment center in his area.
- 1 is waiting to do an internship after the end of a training course carried out within an IT company.

## Discussion

Our project represents an innovative model of intervention because it combines a rehabilitation and treatment approach alongside with job placement. Indeed, it was based on the eight IPS principles: eligibility based on client choice, focus on competitive employment, integration of mental health and employment services, attention to client preferences, work incentives planning, rapid job search, systematic job development, and individualized job supports (Bond, Drake & Becker, 2012; Drake, et al., 2012). Additionally, the innovative perspective is not to try to change individuals’ skills through a pre-work training to better fit with work environment but to find a good match between personal interests and skills and a good workplace wherein the right amount of support can be given (Rinaldi, Perkins, Glynn, Montibeller, et al., 2008). Hence our project considers both the enhancement of social skills and the cognitive abilities to support the employment of ASD people. As highlighted by Baker-Ericzén et al. (2018), these are main aspects for ASD job inclusion and maintenance. A further innovative aspect was the integration of social and cognitive skills enhancement into a “*Place and Train*” paradigm: cognitive and social skills training were conducted in parallel with internships.

The results obtained in the cognitive tests show statistically significant changes for inhibitory control and visuo-spatial and constructive abilities. These results are in line with the objectives of the training while the improvement in visuo-spatial and constructive skills could be partly due to a reduced impulsivity in the response style. Further investigations are needed to validate these hypotheses.

No statistically significant changes were detected in the psychological tests. However, as far as the social domain is concerned, the absence of significant post-training results could be due to an increased awareness of the participants’ dysfunctional modalities which, before the intervention, they acted unintentionally. In our opinion, receiving feedback on unaware behaviors from the psychotherapist or other participants could have improved social awareness and consequently a pejorative evaluation of one’s own behavior.

The use of a feedback questionnaire was a useful and inclusive choice which allowed the participants to clearly express their point of view by providing hints and suggestions for future projects.

Furthermore, the evaluation of the effectiveness of our model can be assumed from the rate of adherence to the internships (4 out of 5 of the funded internships) and the employment status of the participants (7 out of 10), as highlighted at the end of the project.

Case-series study includes methodological limitations due to the small sample size and limited generalization of trainings’ results. In future research, several limitations can be addressed: increase the duration of the project so of the trainings, other-report (parents or caregivers) measures should be included, ensure a major number of paid internships for the entire sample.
